# A scoping review on pair housing dairy calves: health and performance outcomes and tactics to reduce cross-sucking behavior

**DOI:** 10.3389/fvets.2025.1568164

**Published:** 2025-06-27

**Authors:** Gillian D. Plaugher, Melissa C. Cantor

**Affiliations:** Department of Animal Science, Pennsylvania State University, University Park, PA, United States

**Keywords:** social housing, calf, diarrhea, animal welfare, validated clinical scoring, bovine respiratory disease

## Abstract

Calves raised in pairs or triplets often experience better growth performance outcomes when compared to their individually housed peers. However, veterinarians may be concerned that pair housing compromises calf health, and producers are concerned about abnormal oral behavior (e.g., cross-sucking). In this literature review, we evaluated the effect of pair or triplet housing vs. individual housing practices on calf health outcomes and performance since 2016. We also evaluated the literature on mitigation strategies to ameliorate cross-sucking in socially housed calves. We found that when researchers used pair housing practices, there was a lack of association between housing practice and risk of bovine respiratory disease (BRD) status in all studies (100%, 7/7). Only one study lacking healthy control calves found a negative effect on calf diarrhea in week 3 (1/8 studies). However, a moderate number of researchers (57%, 4/7) did not use a validated clinical scoring system to diagnose calves with BRD status. Half of the researchers (50%, 4/8) also did not report their diagnostic criteria for diagnosing diarrhea in their calves, and we suggest this is needed for future work. All researchers who fed calves at least 7 L/d of milk and recorded calf starter intakes found that pair-housed calves consumed more calf starter either preweaning or post-weaning (100% 6/6). However, growth benefits were only observed in 4 studies, in which 75% fed calves at least 7 L/d of milk. Cross-sucking is mitigated by providing socially housed calves with an outlet for oral behavior, such as a teat for milk feeding, offering at least 7 L/d of milk, offering a teat with starter, and forage. We recommend that future studies investigating social housing utilize validated clinical scoring systems for calf health monitoring, report disease diagnostic criteria, and feed ≥ 7 L/d of milk to promote performance benefits in pair-housed calves. More research is needed to understand how cross-sucking develops as a habit in socially housed calves.

## 1 Introduction

Dairy producers adopted individual housing for calves over 40 years ago when veterinarians were concerned about social housing practices promoting the horizontal transmission of diseases among calves (1). However, surveyed dairy producers recently stated that social housing was not viewed as a negative factor for calf health, and that the practice was favorable for calf welfare and social development (2). This is likely because there is a myriad of evidence that pair housing practices for dairy calves improved social cognition and learning ability, positively impacted cognitive judgment bias, and did not compromise calf performance (as reviewed by (3)). While the previous review highlighted the importance of social housing for cognitive development, performance, and calf behavior, several factors were not extensively discussed, including the effects of social housing on calf health outcomes. It is important for veterinarians to have access to a literature review that summarizes the association of pair housing with calf health outcomes because some producers are required to adopt pair housing due to processor mandates (i.e., Tesco in the United Kingdom), or because of the perceived benefits for the calf. Moreover, cross-sucking, an abnormal oral behavior where a calf suckles on another calf, is considered a negative factor for producers considering the adoption of social housing (2). Cross-sucking occurs in pair-housed calves (4). There is some evidence that cross-sucking may increase the risk of mastitis in pair-reared calves (5), though others found no association of cross-sucking with long-term udder health (6). Thus, it is equally important for veterinarians to have access to a review that covers management strategies to mitigate cross-sucking in calves. The impact of different milk feeding levels in socially housed calves is also not addressed in the previous review. The plane of milk nutrition needs to be considered for pair housing since nutritional guideline updates were recently released (7). Since 2016, a wealth of literature has emerged about the effects of housing practices on the risk of calf diarrhea (8 studies), bovine respiratory disease (BRD; 7 studies), and performance (15 studies). This review is warranted because an update on the most recent literature is necessary to continue promoting the adoption of social housing for dairy calves, as individual housing is still standard practice in the United States.

The objective of this literature review was to review the effect of pair or triplet housing practices on calf diarrhea and BRD outcomes, and performance in studies published since 2016 compared to individual housing practices. We begin with a brief introduction to each topic of interest, then discuss the current literature and make recommendations for future research. Since the last published review about the effects of pair housing on calf performance (3), 15 additional studies have been published. Recent updates to the nutrient requirements for calves (7) have led many researchers to feed a higher plane of milk (hereafter referred to as ≥ 7.0 L/d milk) to calves. Highlighting the outcomes in pair-housed calves fed higher planes of milk is necessary to promote proper nutritional management for calves. We will also evaluate the effects of social housing on calf health outcomes because these studies have been published in the past 10 years and are necessary to promote social housing to veterinarians. Thus, we will also review management factors that mitigate cross-sucking behavior in dairy calves for veterinarians to make better-informed recommendations to their clients, considering the adoption of pair housing.

## 2 Eligibility criteria

We included studies for this literature review if the researchers evaluated the effect of pair or triplet housing practices on calf diarrhea outcomes, BRD outcomes, or performance outcomes (average daily gain (ADG), body weight, or calf starter intake), since the last literature review on the effects of social housing on calf performance (3). We included studies that followed calves in the preweaning period to evaluate the volume of milk fed per day as a management factor associated with performance outcomes. More complex group housing was not included in this review because the transition from individual to pair housing may be easier for producers to implement than housing calves in larger groups, which usually requires barn modification. We retained any study that labeled calves as “pairs” including one study that used triplet housing vs. individual housing to investigate the effect on calf health outcomes. We also included a review of management strategies that ameliorated cross-sucking, as this behavior is an issue in socially housed calves and may be viewed as a barrier to the adoption of social housing by producers.

### 2.1 Databases and search terms

**Figure 1:**
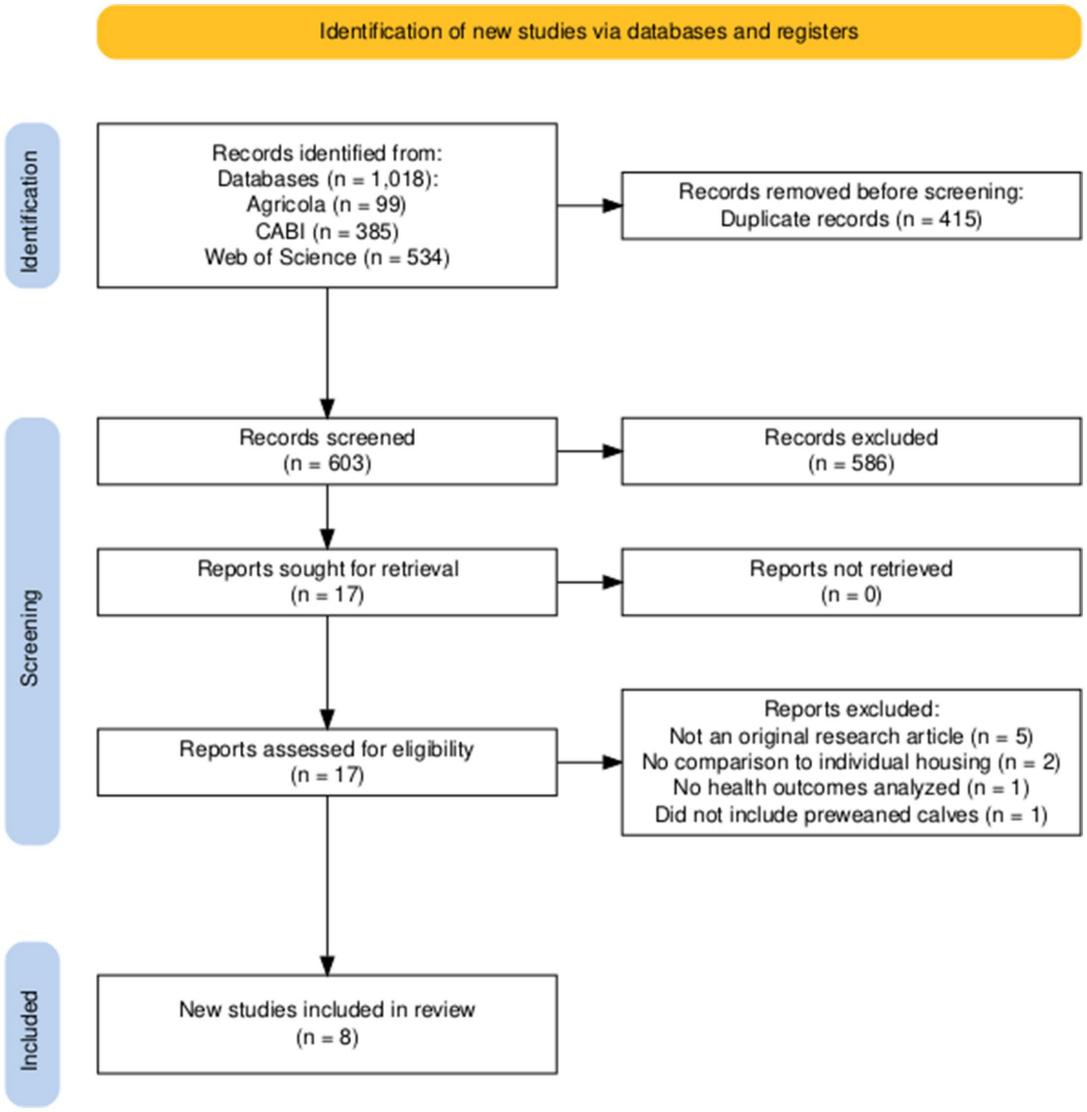
A PRISMA flowchart (92) illustrating the methodology used to identify articles for inclusion in this literature review investigating pair and triplet housing of dairy calves and health outcomes since 2016.

**Figure 2:**
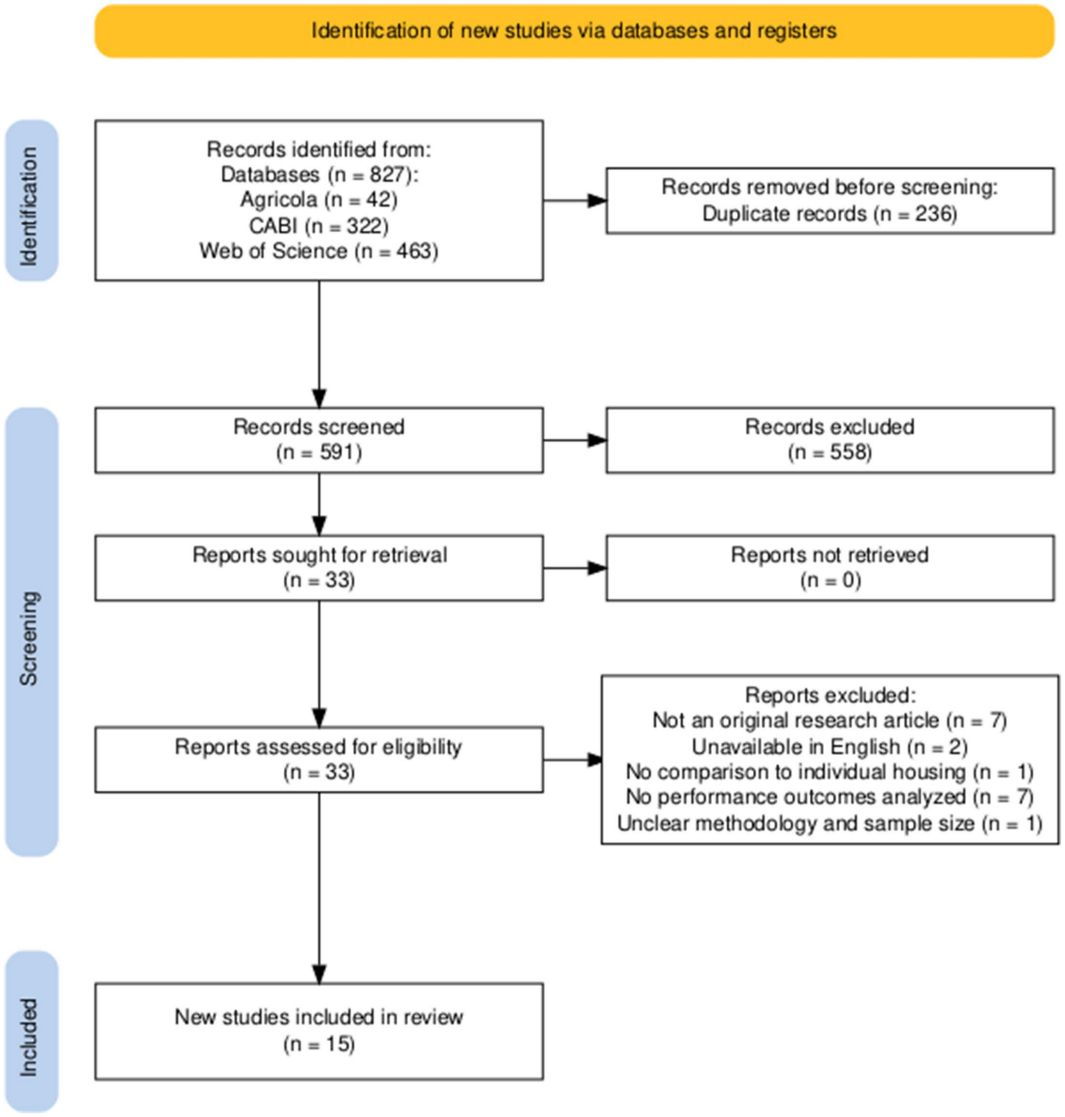
A PRISMA flowchart (92) illustrating the methodology used to identify articles for inclusion in this literature review investigating pair and triplet housing of dairy calves and growth performance outcomes since 2016.

The literature search was conducted using the following databases: Web of Science, CABI, and Agricola. We used Boolean operators within search strings (i.e., AND and OR). An initial search for the effect of pair housing on calf health outcomes was performed using the following search string: pair AND calf AND health OR pair AND calf AND diarrhea OR pair AND calf AND respiratory OR pair AND calf AND disease OR pair AND calf AND mortality. A depiction of the study selection diagram and filtering process for the effect of pair housing on calf health outcomes is in (Figure 1). An initial search for the effect of pair housing on calf performance outcomes including average daily gain, grain intake, and body weight was performed using the following search string: pair AND calf AND performance OR pair AND calf AND growth. A depiction of the study selection diagram and filtering process for the effect of pair housing on calf performance outcomes is in (Figure 2). We filtered all publications by year (since 2016), then extracted the research title and doi from each article into an excel file. The duplicate titles were removed by filtering in excel. Abstracts were screened in each article for pair or triplet housing management practices, then filtered if the outcome reported in the abstract was related to calf performance, BRD, or diarrhea. Finally, the first author re-checked each article for performance and health outcomes at the full-text level to confirm that the comparison group was individually housed calves.

**Figure 3:**
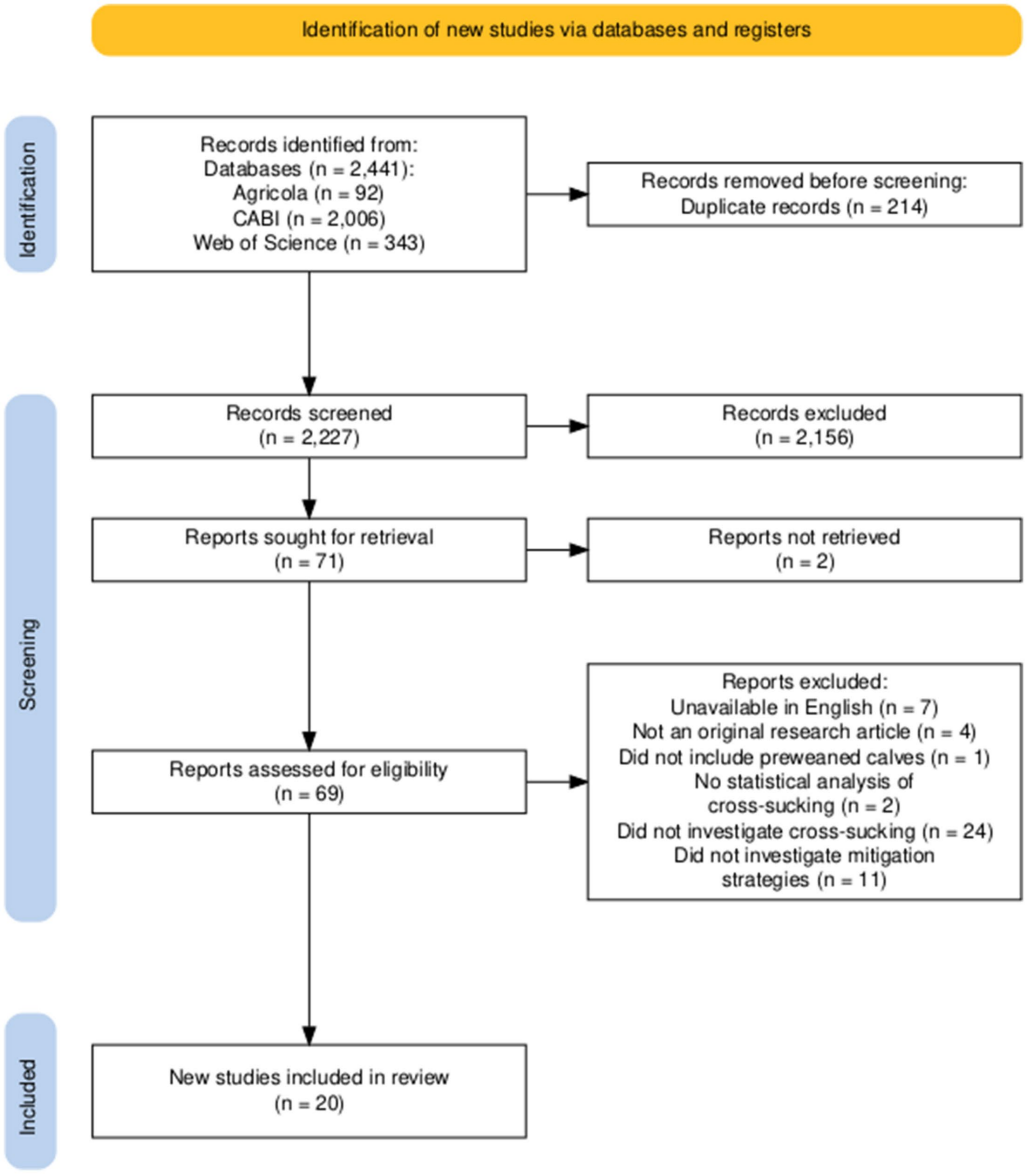
A PRISMA flowchart (92) illustrating the methodology used to identify articles for inclusion in this literature review investigating strategies to mitigate cross-sucking in socially housed calves.

An initial search for tactics to reduce cross-sucking was performed using the following search string: cross-sucking OR calf AND abnormal behavior OR calf AND stereotypy. A depiction of the study selection diagram and filtering process for tactics to reduce calf cross-sucking behavior is in (Figure 3). The main author manually removed research studies that did not investigate the effect of a management factor on lowering the likelihood of cross-sucking in preweaned calves by abstract review. All articles were evaluated at the full-text level to determine eligibility. One study was not populated using key search terms for calf health outcomes and was identified during the full-text screening for performance articles (8). This article was included in the calf health outcome part of the review. After filtering, there were 15 studies: 8 that evaluated the effect of pair housing on calf diarrhea, and 7 for BRD outcomes. There were 15 studies that evaluated the effect of pair housing on calf performance since 2016. There were an additional 20 studies that were included in the scientific review regarding tactics to reduce cross sucking in calves. Thus, there are 35 unique studies included in this literature review.

### 2.2 Data extraction

To create the calf health and calf performance tables, specific search criteria was extracted from each article using the title, abstract, and screening of the full text (if the information was not provided in the title or abstract). For calf health outcomes, the descriptive data was extracted into an excel file. This included, year, authors, title, citation, doi, abstract, study sample size, and that the referent was individual housing. Then, more detailed characteristics about the study design were extracted by manual screening of the abstract, followed by the main body of the article if they were not reported in the abstract. This included the stat analysis used to investigate the association of housing type with health outcomes, the inter-observer reliability for health scoring, the mortality rate of the study, and whether a power analysis was conducted. Features about calf health were extracted from the articles, including the type of calf health scoring system used for diarrhea and or BRD, if it was a scientifically validated system, and the disease definition used to classify calves with diarrhea and BRD, respectively. Last, the results were manually extracted as either neutral no association of housing with BRD or diarrhea outcomes, protective (pair housing benefited calf health), or negative (pair housing increased odds of disease). One reviewer requested the country for each study in our table so this was manually added by screening the site of study for each included publication.

For calf performance outcomes, descriptive data was extracted into an excel file. This included, year, authors, title, citation, doi, abstract, study sample size, and that the referent was individual housing. Then, more detailed characteristics about the study design were extracted by manual screening of the abstract, followed by the main body of the article if they were not reported in the abstract. This included if milk feeding level was at least 7.0 L/d, and if the outcomes included either average daily gain, daily live weight gain, calf starter intake, and or body weights. Last, the results were manually extracted as either neutral (no association of housing with the performance outcomes of interest), protective (pair housing benefited calf performance), or negative (pair housing compromised a performance outcome).

For cross-sucking outcomes, first descriptive data was extracted into an excel file. This included, year, authors, title, citation, doi, abstract, housing type, and study sample size. Then, more detailed characteristics about the study design were extracted by manual screening of the abstract, followed by the main body of the article if they were not reported in the abstract. This included mitigation strategies surrounding cross-sucking, and finally, the results were extracted as either neutral (no association), positive (increased cross-sucking behavior), or negative (decreased cross-sucking behavior).

## 3 Calf health

### 3.1 Morbidity and mortality

According to a USDA survey, the producer reported preweaned heifer calf morbidity rate for calves in the United States is 34%, and most morbidity can be attributed to diarrhea and BRD [21% diarrhea, 13% BRD; (9)]. Morbidity rates are difficult to compare across regions and studies due to a lack of standardized definitions for clinical disease in calves. In pair housing studies, morbidity rates varied greatly, as some studies observed morbidity rates for diarrhea at 11% (10), which is lower than USDA survey data (9), while others observed 100% incidence (11). An incidence of 100% is concerning because a lack of healthy controls limits our ability to claim that the exposure (the housing practice) is associated with the outcome (disease). We suggest researchers use case–control study designs when evaluating calf health as an outcome because this study design helps to evaluate if there is an association between an exposure and a specific health outcome (12). Reporting morbidity and mortality rates in studies measuring health outcomes is also imperative because it indicates disease pressure in the dairy herd to help readers understand the external replicability of the research. Diarrhea and BRD are issues for producers who want to implement pair housing, and any research evaluating calf health should report morbidity in their studies so that the industry may make informed decisions about the disease pressure in the facility.

Another issue we observed was a lack of reporting of mortality rates in the research. Researchers must report mortality rates in calf research because, excluding deaths within 48 h of birth, the 5% mortality rate for preweaned calves in the United States was attributed mostly to diarrhea (56%) and BRD [24%; (9)]. Similar mortality rates for preweaned dairy calves have been observed in other countries, with Great Britain at 5% for calves under 3 months of age (13), Canada at 6% (14) and Finland at 6% for calves 6 to 180 days postnatal (15). A few research publications, 50% (4/8) reported mortality outcomes (Table 1), suggesting room for improvement. For example, Mahendran et al. (16) reported a mortality rate of 2%, Bolt et al. (17) had a mortality rate of 3%, and Mahendran et al. (10) reported a mortality rate of 3%, which are all lower than national averages across the world. We suggest that researchers need to report mortality rates when evaluating the effect of housing on calf health outcomes for transparency, to avoid reporting bias, and to include loss to follow-up for observational research per the guidelines recommended by veterinarians (18). Future studies confirming that pair housing does not have a negative impact calf mortality rates on farms are necessary for the continued adoption of social housing.

**Table 1:** Studies (*n* = 8) since 2016 comparing pair (P) or triplet housing (T) vs. individual housing (IN) on the effect of calf diarrhea and or bovine respiratory disease outcomes.

Authors	Sample size	Statistical analysis	Diarrhea		Bovine respiratory disease	
			Housing effects^1^ Pair referent	Validated scoring? Disease definition?	Housing effects^1^ Pair referent	Validated scoring? Disease definition?
Pempek et al. (8)	20 IN 10 P	Average	=	(61) ≥3	NA	NA
Bolt et al.^2^ (17)	8 IN 16 P	One-way MANOVA	=	No Not reported	=	Yes UW Madison
Liu et al.^3^ (28)	10 IN 10 P	Chi-Square test	+	Larson et al. (25) > 2	NA	No Nasal, breathing difficulties, rectal temp ≥39.5\u00B0C
Bučková et al. (45)	22 IN 22 P	Mixed linear models	=	No Not reported	=	No Frequency of Respiratory Problems
Mahendran et al.^4^ (16)	20 IN 20 P/40 IN 20 P	Binary logistic generalized estimating equations	= Overall disease	No Not reported	= Overall disease	No Nasal, ocular, cough, rectal temp ≥39.5\u00B0C
Mahendran et al. (10)	150 IN 150 P	Logistic Regression	- Overall disease	No Not reported	- Overall disease	No Not reported
Abdelfattah et al. (11)	21 IN 7 T	Cox hazard, Fischer exact	=	Feldmann et al. (105)	=	Yes UC Davis
McFarland et al. (30)	46 IN 46 P	Mixed linear models	=	Yes Yes	=	Yes UC Davis

^1^Effects of pair housing on outcomes: (=) no association of housing with disease, (+) increased probability of disease for pair housing, (−) decreased probability of disease for pair housing. ^2^8 P paired at 5 d; 8 P paired at 28 d. ^3^Increased disease only during week 3 of study. ^4^20 P paired after 3 weeks of individual housing.

### 3.2 Diarrhea

The most common disease that affects preweaned dairy calves is diarrhea (9). A calf has diarrhea when the water content of the feces increases, and the fecal dry matter decreases to approximately 17% or lower on average (19). Calves experience watery diarrhea because of infection, abrupt nutritional changes to the diet, or a combination of these factors (20). Most calves experience diarrhea within 1 to 21 days of age (21). It is important to consider that producers may underreport the incidence of diarrhea, as researchers who followed the fecal consistency of calves daily observed up to 90% of calves becoming sick (22–24). Therefore, the rate of diarrhea reported by producers in the USDA survey may be the number of calves that required intervention on their farm (9). We suggest that more research is needed to identify what the average incidence rate of diarrhea is in dairy farms in North America so that comparisons can be made within the literature.

#### 3.2.1 Fecal consistency scoring: validated clinical scoring systems

When evaluating the current literature, we found it concerning that no researchers reported using a validated clinical scoring system to assess the effect of pair housing on calf diarrhea outcomes (Table 1). Of the pair housing studies that reported the effects of housing treatment on fecal consistency, 50% (4/8) did not define the criteria for their fecal scoring systems, and the other 50% cited unvalidated fecal consistency scoring systems (3/8) or created their own (1/8). Larson et al. (25), called for a standardized fecal consistency scoring system in dairy calves almost 50 years ago, suggesting this has been a need in research for many years. To our knowledge, only Renaud et al. (19) validated a fecal consistency system to assess calves for diarrhea. They observed that when a calf is rectally stimulated to defecate, an increase in fecal consistency score had a strong negative correlation with a decrease in fecal DM, indicating diarrhea (19). A scale of 0 to 3 was used following Larson et al. (25): “0 = normal, firm but not hard, original form is distorted slightly after dropping to floor and settling; 1 = soft, does not hold form, piles but spreads slightly, 2 = runny, spreads readily, and 3 = watery, liquid consistency, splatters” (19). We acknowledge that half of the reviewed studies 43% (3/7), were published before Renaud et al. (19). However, it is fundamental that researchers do not use tail scoring, or wet calf perineal area to observe for calf diarrhea as it was not validated in a recent study (26). We suggest researchers should only use Renaud et al. (19) for fecal consistency scoring calves for diarrhea in future work, as it is the only validated system.

#### 3.2.2 Case definition for calf diarrhea

Recently, the lack of standardized definitions for diarrhea in calf research was highlighted as an issue in a scoping review (27). Only half the researchers (50%; 4/8) that evaluated pair housing effects on calf diarrhea outcomes reported their case definition. Only one researcher (1/7) identified an increased risk of diarrhea with pair housing at week 3 of life, but this study lacked healthy control calves (28). Liu et al. (28), also used more than one system including Larson et al. (25) for fecal scoring consistency scoring, and wet feces on the tail or hindquarters of a calf, which is not a valid scoring system for evaluating calves for diarrhea (26). Thus, we suggest that the one study that found a negative effect of social housing on week 3 of life related to diarrhea risk likely had additional observed cases since they used an invalidated diarrhea scoring system. We suggest that it is fundamental for researchers to report their case definition for diarrhea, especially because the disease definition for calf diarrhea varies among studies. It is also important that researchers use one standardized definition for diarrhea to avoid variability. Researchers must use appropriate case definitions in future work where calf health is used as an outcome.

#### 3.2.3 Frequency of fecal consistency scoring

The frequency of fecal consistency scoring is important for calf health research because infrequent scoring leads to missed diarrhea cases. The duration of diarrhea in calves varies and can be as short as 1 day and as long as 12 days (20). This is because the duration of calf diarrhea varies by pathogen (29) or can even vary when calves are sourced from multiple farms (22). Half (50%; 4/8) of the studies that evaluated the effect of pair housing on diarrhea evaluated calf fecal consistency daily, but 25% (2/8) scored calves once a week, suggesting cases of diarrhea were missing in those studies. Others, such as McFarland et al. (30) did not report how frequently calf diarrhea was assessed, relying on commercial dairy producer records. A highlight of this review was that all studies evaluated calves during the critical period for susceptibility to pathogenic diarrhea, from 1 to 21 days of age (29). We suggest that future research should adopt daily fecal consistency scoring in calves to avoid missing cases of the disease, but this was a strength that occurred in most of the studies evaluated. In summary, we suggest that pair or triplet housing did not increase the risk of calf diarrhea.

### 3.3 Bovine respiratory disease

The second most common disease affecting preweaned calves is Bovine Respiratory disease (9). Bovine Respiratory Disease is an infection of the respiratory tract in cattle which can be viral, bacterial, or a co-pathogenic infection (31). An important consideration is that BRD may be underdiagnosed in some studies, as researchers who used thoracic ultrasound observed more calves with lung consolidation than cases of BRD observed by producers using only outward signs of disease to diagnose their calves (32). However, a large population-wide study of over 10,000 calves observed an incidence rate of BRD (e.g., 15%) that was very similar to the rate reported by producers in the USDA survey (33). Evidence that pair housing does not increase BRD rates in calves is needed to ensure that BRD is not viewed as a barrier to entry by producers and veterinarians.

#### 3.3.1 Validated clinical scoring systems

Unfortunately, we found that only 43% (3/7) of the studies that evaluated the effect of pair housing on BRD in calves used a validated clinical BRD scoring system or reported a disease definition (Table 1). Fundamentally, researchers must report their case definitions for BRD, as disease diagnostic protocols are linked to antimicrobial intervention strategies for BRD on dairy farms (34). Specifically, Abdelfattah et al. (11) and McFarland et al. (30) used the UC-Davis system and Bolt et al. (17) used the UW-Madison System. These studies observed no association of pair housing on the probability of BRD in calves. All researchers (100%; 7/7) found no association of pair housing with the probability of BRD status in the calves. Researchers that used validated clinical BRD scoring systems evaluated calves for a combination of outward signs of BRD such as cough, ear tilt, cloudy nasal or ocular discharge, rectal temperature, and heavy respiration rate (as reviewed by (35)). These clinical BRD scoring systems include the UW-Madison System (36), the UC Davis System (31), and the use of thoracic ultrasound scoring (TUS) to evaluate calves for internal lung consolidation (37).

Validated clinical BRD scoring systems are commonly used to evaluate calf health outcomes. In fact, the sensitivity of these systems is moderate (62 to 78%), and the specificity is moderate to very high [74 to 91% (32, 38, 39)]. Thus, we suggest that outward clinical BRD scoring likely overdiagnoses BRD in calves. A study observed similar diagnostic ability between the UW-Madison and UC Davis system to identify BRD in calves, suggesting that these systems have similar diagnostic capabilities (40). In agreement, others who used a non-validated BRD scoring system observed no association of pair housing with BRD, but interpretations are limited because the systems were not scientifically validated. However, one research group recently observed no association of outward signs of ocular and nasal discharge, and ear tilt with lung consolidation in calves, suggesting these individual outward signs of disease may not be appropriate diagnostic criteria to define cases of BRD in calves (41). Future research is needed to develop additional systems for researchers to use to identify outward signs of BRD in calves. However, we note that no study found any association of pair housing with increased risk of respiratory disease status.

Researchers use thoracic ultrasound scoring (TUS) to observe calves with lung consolidation, when the lung lacks reverberation artifact, appears hypoechoic and lacks the pleural surface (37). Thoracic ultrasonography scanning calves for BRD is as sensitive as radiography which has a sensitivity (Se) of 89% and specificity (Sp) of 58% (42), suggesting TUS is the best point-of-care option for field research in calves. Specifically, TUS ranges from moderate to very high Se 74 to 94%, and moderate to perfect Sp 74 to 100% (32, 37, 42, 43). In contrast, the validated clinical BRD scoring systems that were used to score calves in pair housing research have low to moderate Se (62 to 78%) and moderate to very high Sp (74 to 91%) (32, 38, 39).

We found that 29% (2/7) of studies that evaluated the effects of pair housing on BRD in calves used TUS; these studies did not observe an association of pair housing with the probability of respiratory disease (10, 16). However, these researchers only scored the calves once at weaning, limiting study findings. Cross-sectional research should not be used when evaluating the effect of a management factor on BRD in calves because this study design is used to determine the prevalence of disease rather than the overall incidence rate (44). Instead, a case–control study is appropriate as disease occurrence can be followed throughout time (18), and we can evaluate for an association between exposure (housing) and the health outcome (12). We suggest that researchers implement TUS for point-of-care diagnostic research when BRD status is an outcome of interest because it is diagnostically accurate.

#### 3.3.2 Case definition for bovine respiratory disease

We observed that some researchers (43%; 3/7) used a validated definition of clinical BRD when evaluating the effect of pair housing on the probability of BRD in dairy calves, these were the same researchers who used a validated clinical BRD scoring system (11, 17, 30). For example, Bučková et al. (45) monitored calves for outward signs of coughing, ocular and nasal discharge, and hampered respiration, assigned a score of Y or N, and then reported BRD outcomes as “frequency of respiratory problems.” Liu et al. (28) evaluated calves for nasal discharge, and breathing difficulty via auscultation or cough, then evaluated if a calf needed treatment for fever. The use of invalidated BRD scoring systems is concerning, and this has been previously observed in human medicine in a systematic review of chronic obstructive pulmonary disease (COPD) prognostic indices. The review found that several factors known to be prognostic indices of COPD were not included in many studies and there were many predictors leading to low uniformity of the studies overall (46). While human respiratory health is not directly comparable to BRD in calves, it suggests that researchers often rely on veterinary protocols rather than using validated clinical BRD scoring systems to ensure reproducibility. Our overarching recommendation for future calf health research is that researchers use validated BRD clinical scoring systems, and ideally, TUS to ensure that calves labeled with BRD are clinically sick.

### 3.4 Study design factors in calf health research

#### 3.4.1 Inter-observer agreement

It is important to evaluate for inter-observer agreement in calf health research to ensure precise diagnosis of disease. Inter-observer reliability is the level of agreement between multiple observers when reporting a subjective outcome and is an important factor to consider when conducting research utilizing more than one observer, although it is often not calculated or reported (47). Commonly used coefficient measurements include Cohen’s kappa (48) for two observers and Fleiss’s kappa (49) for three or more observers. These coefficients provide statistical evidence of the level of agreement as well level of error due to chance between observers independent of training or prior knowledge (50), increasing the validity of findings when using multiple observers. Nearly every study, 88% (7/8), failed to report either the number of observers or the inter-observer agreement for multiple observers when evaluating the effect of pair housing on health outcomes. The one study that met these criteria used three trained observers and reported an inter-observer agreement of 95%, indicating a very high level of agreement, but this study lacked healthy controls (11). The findings of Abdelfattah et al. (11) had inter-rater agreement that was like other research that evaluated calves for diarrhea, (e.g., over 90%) suggesting repeatability (19, 22). Other studies such as Pempek et al. (8) and Bučková et al. (45) did not describe the number of observers, or the inter-rater agreement. Some studies only relied on producer records (30). Observational scoring such as for abnormal eye or nasal discharge varies widely between observers depending on prior knowledge or training (51) which is why Cohen’s or Fleiss’ kappa are so important to calculate for animal health studies. Observer bias, which occurs when one observer may record more observations than another, can be adjusted for if agreement has been calculated (47), reducing the potential for inaccurate statistical findings. The calculation of inter-observer agreement is considered a gold standard for the evaluation of animal behavior, evaluation of human medical criteria and images and we suggest this needs to be required for calf health research (52–55). We highly recommend calculating and reporting inter-observer agreement in any study that reports observational findings including more than one observer.

#### 3.4.2 Power analysis

Another factor affecting statistical outcomes is sample size calculation and power analyses. While this is true for all hypothesis testing research, it is also important for observational calf health research because many studies were likely underpowered for the differences in small effects that they were measuring; this requires a much larger sample size to detect a difference that is not due to random chance (56). Too few animals included in a trial can result in not detecting a statistical difference when there is one, and this could have consequences if an inadequately powered calf health trial does not find a difference among housing treatments, as disease outcomes affect farm profitability (57). Conducting power analyses to account for the biological effects of the sample population is extremely important in animal research (58). We speculate that it is often not reported due to limited availability of animals, funding, or time, but for calf health research it is needed. We acknowledge that some exploratory research studies may not have sufficient literature on the outcomes of interest to conduct a formal power analysis, however best efforts should be made to report how the sample size was determined. A moderate number of studies (63%; 5/8) reported power analyses to determine if their sample size was large enough to detect statistical differences, suggesting many of these studies were convenience sample sizes. Calf health studies must use a power analysis because producers are adopting social housing under the pretense of research findings suggesting that this practice does not negatively affect calf health outcomes. We recommend that future studies which investigate the effects of housing on calf health conduct a power analysis to improve the accuracy of statistical results.

## 4 Calf performance

There is a large amount of evidence that feeding a higher plane of milk nutrition (e.g., more than 7 L/d) improved calf starter intake in pair-housed calves (100%, 6/6; Table 2). The majority (73%; 11/15) of the work conducted since 2016 also offered calves a higher plane of milk nutrition. Many researchers offered calves more milk to reflect the new NASEM guidelines to ensure calves double their body weight by weaning even under cold stress (7). We suggest that pair or triplet housing dairy calves had no negative effects on performance (100%; 15/15). Thus, our findings agree with earlier literature (3). However, it is less clear why some researchers found positive growth benefits in pair-housed calves (59, 60), and many others did not. Only one study that fed calves less than 7 L/d observed growth benefits in pair-housed calves (8) compared to individually housed calves (Table 2). Thus, we suggest that improved performance is likely only observed in paired calves when they are fed more milk.

**Table 2:** Studies (*n* = 15) investigating the effects of pair (P) or triplet (T) vs. individual (IN) housing on performance outcomes in dairy calves since 2016.

Authors	Sample size	Country	≥ 7 L/d milk^1^	ADG/DLWG	Starter intake	Body weights
Pempek et al.^2^ (8)	20 IN 10 P	USA	No	=	+	+
Bolt et al. (17)	8 IN 16 P	UK	No	=	=	NA
Wormsbecher et al. (93)	12 IN 12P	Canada	Yes	=	NA	=
Overvest et al.^2^ (94)	10 IN 9 P	Canada	Yes	NA	+	NA
Whalin et al. (95)	14 IN 8 P	Canada	Yes	NA	+	=
Liu et al.^2^ (96)	10 IN 10 P	China	Yes	=	+	=
Bučková et al. (45)	18 IN 21 P	Czech Republic	Yes	=	+	=
Knauer et al.^2^ (59)	12 IN 6 P	USA	Yes	+	NA	+
Mahendran et al.^2^ (16)	40 IN 20 P	UK	Yes	=	+	NA
Zhang et al. (80)	48^3^	UK	No	=	NA	NA
Mahendran et al. (10)	150 IN 150 P	UK	No	=	NA	NA
Abdelfattah et al. (11)	21 IN 7 T	USA	Yes	=	NA	=
Reuscher et al. (67)	16 IN 16 P	USA	Yes	=	+	=
Riesgraf et al.^4^ (60)	15 IN 13 P	USA	Yes	=	NA	+
McFarland et al. (30)	46 IN 46 P	UK	Yes	=	NA	=

^1^Fed ≥ 7 L/d at any point during the study period. ^2^Treatment × time interaction present. ^3^48 calves split into 8 blocks of 6 calves each, split into IN or P housing, with half of each housing treatment being either physically enriched or not physically enriched. ^4^Paired heifers weighed more at the beginning of the trial than IN heifers (*p* = 0.03), with p = 0.05 at end of study. Increased (+), decreased (−) or similar (=) performance outcomes in comparison to individually housed calves are noted.

It was observed that feeding higher planes of milk in calves promotes growth regardless of the housing practice (61–63). These growth benefits in young calves were attributed to increased milk DM intake and more ME for the calf to grow. Improved ADG during the preweaning period was also associated with an increased first lactation milk production by 3% regardless of housing system (64), so feeding a higher plane of nutrition during this period can have lasting impacts on milk production as well. However, only 18% (2/11) of the studies that fed at least 7 L/d of milk to paired calves observed improved ADG or body weight gain compared to individually housed calves (59, 60). It is unclear why this is, although we hypothesize it could be that pair-housed calves expend more energy on play and social activity (3), therefore maintaining similar growth rates compared to individually housed calves. On the other hand, every study that fed more milk and measured calf starter DMI (6/6) observed an improved calf starter intake either preweaning, and or during the post-weaning period. We suggest that socially housed calves may have improved calf starter intakes due to social facilitation. Social facilitation is well-studied in calves, calves benefit by observing a behavior being performed by peers because it stimulates the behavior in the observer (3). Dairy cattle are highly neophobic to new feeds, and providing exposure to complex social housing in early life is a known social facilitator that decreases the latency for cattle to consume novel feedstuffs (65). Calf starter intake is fundamental for ruminal development because the consumption of concentrate promotes the breakdown of calf starter by ruminal microbes, increasing the presence of butyrate in the rumen, which facilitates ruminal papillae growth (66). However, we suggest that future researchers need to identify if improved calf starter DMI in pair-housed cattle results in greater ruminal papillae development as this has not yet been explored. This has yet to be quantified in socially housed calves and is an important scientific question to answer to promote the adoption of social housing for dairy producers.

Many studies which fed higher milk allotments found no effect of pair housing on calf growth, though some of these were conducted during colder parts of the year (30, 45, 67). Notably, two of these studies were conducted during the winter months where the average temperature was below 0°C, and season is a known factor for affecting growth patterns in calves (68). A dairy calf’s thermoneutral zone (TMZ) ranges from 15 to 25°C (69), suggesting that these two studies were well below the TMZ for young calves (45, 67). It is likely that growth patterns did not differ among socially housed and individually housed calves in these studies because of cold stress (45, 67). However, it is important to note that pair-housed calves spent more time together during cold stress, and the hutch temperature was warmer when pair-housing calves (67). Thus, we suggest that pair housing not only provides performance benefits to calves but that there are also benefits to keeping each other warm during colder conditions.

It is less clear why other studies that were conducted across all seasons observed no benefit to pair housing for calf growth. It is possible that management factors such as colostrum management, sanitation, and other farm factors affected the growth patterns observed in these calves. However, more research is needed to identify why growth benefits are observed in some research studies for pair-housed calves and not others. However, we still recommend pair housing for calf performance because promoting calf starter intake is fundamental in calves offered higher milk allotments.

Few studies have evaluated the long-term benefits of socially housing calves, but most have observed no negative effects of social housing. For example, Riesgraf et al. (60), observed that pair-housed dairy calves-maintained growth advantages into the pubertal heifer period without compromising feed efficiencies or affecting methane emissions. Mahendran et al. (5), observed that pair housing did not compromise health outcomes or affect first lactation milk production. However, the individually housed calves were more likely to exit the herd prematurely (5). On the other hand, Mahendran et al. (5), observed that pair-housed cattle were 93% more likely to have udder issues than individually housed calves, and this was attributed to cross-sucking. We suggest that the biggest barrier to producers adopting social housing is the avoidance of cross-sucking behavior.

## 5 Cross-sucking mitigation strategies

Cross-sucking is an abnormal oral behavior when a calf suckles the underside of another calf (4). It occurs in artificially reared calves as it is not seen in calves raised with their dams (70, 71). Calves cross-suck because they are motivated to drink milk in multiple meals, which differs from the two to three meals most producers provide to their calves. The mechanisms for why this habit starts are poorly understood, though many management factors related to feeding can decrease how often cross-sucking occurs in calves (72). We observed that socially housed calves were more likely to cross-suck when they had less frequent meals, no teat offered, and did not have an outlet such as the provision of forage to perform oral behavior (Table 3). Cross-sucking was recently rated by dairy producers as a negative factor for calf health and welfare, especially in consideration of adopting social housing (2). It is therefore important that researchers identify the mechanisms behind the development of cross-sucking habits in calves. Studies are limited on the long-term consequences of cross-sucking, although some have observed that it is a lifetime habit that increases the risk of culling from the herd (73, 74). Others have observed that cross-sucking is a risk factor for the development of mastitis (75), though these study findings are not consistent across the literature (6). Mahendran et al. (5) also observed that paired calves who cross-suck are more likely to have ear abscesses and navel infections. Thus, there are negative consequences to cross-sucking in calves. When the biological desire to suckle is not fulfilled, calves will suckle on one another to fulfill their need.

**Table 3:** Studies (*n* = 20) investigating management strategies to reduce cross-sucking bouts in socially housed calves.

Author	Sample size	Mitigation strategies	Effect on cross-sucking occurence^1^
Jung and Lidfors (97)	*n* = 33 (11 Triplets)	Milk flow, amount & removal teat	High volume - Teat removal + Milk flow =
Weber and Wechsler (98)	*n* = 29 (15 open stall, 14 closed stall)	Closed door after entry on automatic feeder	-
Jensen and Holm (99)	*n* = 96 (6 blocks of 16)	Milk amount and flow rate	=
Margerison et al. (85)	*n* = 48 (12 blocks of 4)	Cow-suckling	-
Jensen and Budde (76)	*n* = 96 (6 blocks of 16)	Group size, teat vs. open bucket	Group size = Teat bucket -
Fröberg et al. (83)	*n* = 22 (10 cow-suckling, 12 artificially reared)	Cow-suckling	-
Nielsen et al. (100)	*n* = 72 (6 blocks of 12)	Milk allowance & gradual weaning	Increased Milk allowance = Gradual weaning -
Roth et al. (86)	*n* = 27	Concentrate dependent weaning/forage	-
Fröberg and Lidfors (84)	*n* = 41 (23 auto-feeder, 18 suckling)	Cow-suckling	-
de Passillé et al.^2^ (101)	*n* = 32 (8 pens of 4)	Gradual weaning	-
de Passillé et al. (102)	*n* = 45 (5 pens of 9)	Milk allowance, early vs. late weaning	=
Ude et al. (91) ^3^	*n* = 168 (12 pens of 12)	Teats and hay	-
Pempek et al. (77)	*n* = 40 (15 pairs bucket, 17 pairs bottle)	Bottle vs. open bucket	Bottle -
Dong et al. (78)	*n* = 12 (2 pens of 6)	Bottle vs. open bucket	=
Nielsen et al. (103)	*n* = 48 (5 pens of 9–10)	Milk flow & portion	=
MacPherson et al. (104)	*n* = 10 (2 groups of 5)	2 vs. 4 milk meals	=
Salter et al. (4)	*n* = 64 (8 pairs/treatment)	Teat bucket and/or Braden bottle	-
Zhang et al.^4^ (80)	*n* = 48 (Individual or pair)	Brush, chain, teat, and strawberry scented hay	-
Bieber et al. (82)	*n* = 58 (30 bottle, 28 cow suckle)	Cow-suckling	-
Doyle and Miller-Cushon (81)	*n* = 28 (14 Pairs)	Human contact	-

^1^Effect on duration or frequency, (=) no effect on cross-sucking, (−) decreased cross-sucking, (+) increased cross-sucking. ^2^22 d weaning period had highest duration of cross-sucking compared to other treatments during weaning. ^3^Pilot study of 2 pens of 12 calves included in total sample size. ^4^48 calves split into 8 blocks of 6 calves each, split into IN or P housing, with half of each housing treatment being either physically enriched or not physically enriched.

Although mitigation strategies to reduce calf cross-sucking behavior varied widely across studies, providing a teat bucket or bottle to feed milk appears to be the most effective strategy for reducing cross-sucking bouts in artificially reared calves as observed in Table 3. Nearly every study 75% that investigated feeding with a teat vs. an open bucket found that offering a teat decreases cross-sucking (76, 77). Moreover, one group of researchers observed that providing calf starter in a Braden bottle (bottle with a teat) in addition to an open bucket also decreases cross-sucking bouts and duration around milk feeding (4). Thus, it is possible that providing additional suckling outlets may mitigate some cross-sucking bouts in calves. One study found no difference in cross-sucking between calves fed with a bucket or a bottle (78), and this may be due to the feeding plan used in this study. Dong et al. (78) fed calves in their study milk replacer at a rate of 11% of body weight, containing 22% crude protein and 13% fat. We hypothesize that the lower fat content of the milk replacer may not have satiated calves, leading to similar amounts of cross-sucking in both groups of calves. Alternatively, perhaps boredom is a driving factor for cross-sucking in calves as boredom is linked to increased exploratory behavior in calves (79). It is important to investigate which enrichment items decrease cross-sucking in calves because these items improve a producer’s perception of calf comfort (2). Moreover, Zhang et al. (80), found evidence that calf brushes do decrease cross-sucking, though calves spent more time interacting with scented forage than anything else. In summary, feeding calves with teats or bottles can be easily adopted by calf feeders while effectively reducing cross-sucking bouts.

It is important to investigate which strategies decrease cross-sucking in calves because these can improve a producer’s perception of calf comfort in social housing scenarios (2). While it has not been fully explored, perhaps boredom is a driving factor for cross-sucking in calves as boredom is linked to increased exploratory behavior in calves which could promote cross-sucking behavior (79). For example, Zhang et al. (80), found evidence that calf brushes do decrease cross-sucking bouts, though calves spent more time interacting with scented forage than anything else. Human contact was also observed to mitigate cross-sucking behavior in calves (81). However, the most effective option for mitigating cross-sucking behavior is allowing calves to suckle on the dam, as all studies investigating this strategy found decreased cross-sucking frequencies (82–85). This is not surprising since it is known that calves raised naturally with their dams do not exhibit cross-sucking behavior (71), however this strategy could be challenging for producers to implement due to traditional practices on farm.

We observed that offering a higher plane of milk-based nutrition reduces the incidence of cross-sucking bouts (Table 3). The most important factor related to the successful higher plane of milk-based nutrition is offering a gradual step-down weaning strategy to calves to allow time for the gut to transition to calf starter, which decreased cross-sucking bouts in calves (86). Thus, we suggest that paired calves should consume at least 2.8 kg/d of calf starter for at least 3 days before a step-down weaning strategy over at least a week is implemented (7). A step-down weaning strategy decreases cross-sucking bouts by increasing calf starter intakes among pairs. Moreover, there is limited evidence that restricting feed has carry-over effects on cross-sucking bouts in older heifers. One study found that limiting the total mixed ration led to increased inter-sucking among heifers after weaning (74, 87). To overcome this, we suggest that it may be beneficial to offer post-weaned heifers a lower quality forage (<10% crude protein) free choice post-weaning to promote gut fill and avoid hunger.

More complex social housing research has also observed that calves do decrease cross-sucking when forage was offered, but this has yet to be explored in pair housing studies (88–90). Given that many producers are adopting pair housing, we suggest that researchers need to investigate if offering forage to paired calves decreases the frequency of cross-sucking. We observed that some studies presented in Table 3 used forage to mitigate cross-sucking bouts in calves and observed a positive benefit (80, 86, 91). One study observed that an increased duration of foraging decreased cross-sucking in calves regardless of the plane of milk-based nutrition offered (86). However, researchers need to quantify which type of forage is best to offer calves. While each mitigation strategy has pros and cons for producers to consider, we recommend implementing at least one strategy in pair-housed calves to decrease the likelihood of negative effects on calf health and performance due to cross-sucking.

## 6 Conclusion

Pair or triplet housing dairy calves is becoming more popular with producers due to its performance benefits for calves (3), but cross-sucking behavior continues to be a barrier for producers and veterinarians considering the adoption of social housing practices. In this review, we found that pair or triplet housing did not impact calf diarrhea, or BRD outcomes. However, there was little agreement among studies regarding disease definition and reporting, which may have affected findings in these studies. We suggest that a longitudinal study is necessary using validated health scoring systems and more sensitive point-of-care tools (such as thoracic ultrasonography) to confirm that calf health is not compromised by pair housing practices. However, pre-emptive research suggests that cross-sucking behavior is likely a greater barrier to the adoption of pair housing compared to calf health concerns. We highly recommend only utilizing validated clinical scoring systems, and validated case definitions for diarrhea and BRD in all future studies investigating health outcomes for socially housed calves. Reporting inter-observer agreement and providing a power analysis is also not routinely performed in calf health studies. The gold standard for observational research is to report power analyses and inter-observer agreement so we recommend this moving forward, especially for future meta-analyses in this research area.

Pair housing dairy calves was also proven to not negatively affect performance. We observed that when calves are offered at least 7 L/d of milk, all studies found improved calf starter intake and only a few studies observed growth benefits over individual housing. However, further research is necessary to confirm whether pair housing affects rumen development, and intestinal morphology as this has yet to be researched. Most studies offered a higher plane of nutrition to calves to meet the updated nutrient requirements set by NASEM (7). However, some studies still fed lower amounts of milk. We believe that future studies should feed calves ≥ 7 L/d of milk since it is correlated with improved performance in pair housing systems. There are limited performance benefits to pair housing if calves are offered < 7 L/d, thus we discourage feeding limited milk to future pair-housed calves. We also suggest that the long-term effects of pair housing on future lactation performance need to be investigated in North America, as only one study exists in the United Kingdom. Regional differences such as climate may affect pair housing carry-over effects on lactation performance.

We observed that calves cross-suck because they are motivated to drink milk from a teat in multiple meals, which differs from the two to three daily meals that most producers provide to their calves. Since cross-sucking is a top concern for dairy producers (2), it is fundamental that researchers identify why calves start cross-sucking. There is some evidence that offering milk with a teat, and other outlets for oral behavior such as offering a low crude protein forage, calf starter in a teat bottle, and enrichment items decreases cross-sucking, yet none of these items eliminate the behavior. More research is needed to identify the long-term consequences of cross-sucking and to identify ways to inhibit the behavior from starting in the first place.

In summary, we suggest that pair housing positively impacts calf starter intakes and may impact growth performance in thermoneutral conditions when calves are offered at least 7 L/d of milk. There are no negative effects of pair or triplet housing on calf health outcomes such as bovine respiratory disease, or calf diarrhea in studies with healthy controls. Cross-sucking is still a challenge for dairy producers adopting social housing practices and more research is needed to identify nutritional strategies and management strategies to discourage and or eliminate this behavior. We suggest that the performance benefits and lack of health consequences on pair housing practices make this a suitable social housing strategy for dairy producers.
